# A Minimally Invasive Approach for Correction of Anterior Spacing and Proclination With Lithium Disilicate Glass-Ceramic Veneers: A Case Report

**DOI:** 10.7759/cureus.80070

**Published:** 2025-03-05

**Authors:** Vidhi Mall, Ashwini Gaikwad, Sanpreet S Sachdev, Aishwarya Handa, Shivani Chavan, Mineet Kaul

**Affiliations:** 1 Conservative Dentistry and Endodontics, Bharati Vidyapeeth (Deemed to be University) Dental College and Hospital, Pune, IND; 2 Oral Pathology and Microbiology, Bharati Vidyapeeth (Deemed to be University) Dental College and Hospital, Navi Mumbai, IND

**Keywords:** aesthetic dentistry, ips e-max, minimally invasive restoration, porcelain laminate veneer, smile design

## Abstract

Achieving a balance between function and aesthetics is a critical aspect of restorative dentistry, particularly in the anterior maxillary region. This case report presents the use of lithium disilicate glass-ceramic laminate veneers as a minimally invasive solution for a 37-year-old female patient with spacing and proclination of upper anterior teeth. The patient sought an aesthetic enhancement but declined orthodontic treatment. A comprehensive treatment plan, incorporating digital mock-ups and mathematical indices for smile design, was formulated. IPS e-max veneers were fabricated and bonded using an adhesive protocol to enhance durability and aesthetics while preserving natural tooth structure. The final restorations achieved significant improvement in smile harmony, proportion, and function. At a six-month follow-up, the veneers demonstrated excellent stability, with no signs of debonding, discoloration, or gingival inflammation. This case highlights the effectiveness of lithium disilicate glass-ceramic veneers in addressing aesthetic concerns while maintaining minimally invasive principles, ensuring long-term success and patient satisfaction.

## Introduction

In restorative and aesthetic dentistry, achieving the ideal balance between function and appearance is often a primary goal, particularly for patients seeking treatments to address aesthetic concerns. The anterior region of the maxilla plays a critical role in the overall aesthetics of the smile and face. Dental professionals are increasingly faced with the challenge of providing treatments that not only address functional concerns but also meet patients’ high aesthetic expectations. As the demand for minimally invasive treatments continues to grow, the development of advanced restorative materials and techniques has provided clinicians with the means to deliver results that are both durable and highly aesthetic [1].

Lithium disilicate glass-ceramic laminate veneers are among the most favored options for addressing aesthetic concerns in anterior teeth. These restorations offer the advantage of being less invasive, requiring minimal tooth preparation while providing a highly aesthetic outcome [2]. Their ability to mimic natural tooth characteristics, such as translucency and texture, combined with their durability, makes them a reliable choice for patients seeking long-lasting aesthetic improvements. Additionally, the advancements in adhesive dentistry and ceramic materials, such as lithium disilicate, have enhanced the bond strength and structural integrity of veneers, ensuring predictable outcomes [3].

A key consideration in aesthetic dentistry is the proportionality and harmony of the teeth in relation to facial features. Mathematical indices, such as the Golden Proportion and Berry’s Biometric Index, are often employed to guide the design of anterior restorations [4]. These indices help ensure that the width, height, and spatial arrangement of the anterior teeth are aesthetically pleasing and functionally appropriate. Furthermore, comprehensive treatment planning that includes an evaluation of factors such as overjet, overbite, occlusion, and gingival health is essential for the success of any aesthetic treatment.

This case report presents an exploration of the principles of aesthetic dentistry and highlights the clinical application of lithium disilicate glass-ceramic laminate veneers as a minimally invasive and effective solution for addressing aesthetic concerns in the anterior region.

## Case presentation

A 37-year-old female patient presented to the Department of Conservative Dentistry and Endodontics with the chief complaint of spacing between her upper anterior teeth since adolescence. The patient had previously undergone root canal treatment in the upper right posterior region one month prior and reported no significant medical history. Clinical examination revealed inadequate lip closure at rest due to the proclination of the upper anterior teeth. Significant overjet and overbite with the anterior teeth were noted.

A thorough soft tissue examination showed no abnormalities. Hard tissue analysis revealed proclination and spacing in the maxillary central incisors (11, 21) and lateral incisors (12, 22), with distal rotation of tooth 22. The patient exhibited an Angle’s Class I molar relationship with bimaxillary protrusion. Pit and fissure caries were noted in the mandibular molars (36, 46). The patient was advised orthodontic treatment as the optimal solution to correct the alignment and spacing, but she declined due to the time-intensive nature of orthodontic therapy.

**Figure 1:**
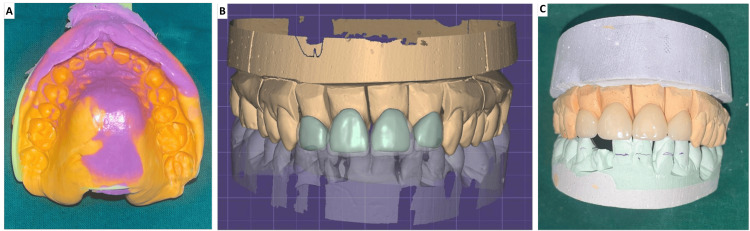
(A) Diagnostic impressions. (B) Digital mock-up planning. (C) Glazed veneer after fabrication.

After discussing various restorative options, including full coverage crowns, indirect partial coverage restorations, and direct bonded composite restorations, the patient expressed interest in a less invasive and more aesthetic solution. Considering the patient’s desire for minimal tooth reduction and long-lasting aesthetic results, lithium disilicate glass-ceramic laminate veneers were recommended as the most appropriate treatment option. The decision to proceed with veneers was based on their ability to correct spacing, improve alignment, and provide superior aesthetics with minimal invasion. The treatment began with pulp vitality testing using an electronic pulp tester for teeth 13-23, confirming the vitality of all the teeth. Diagnostic impressions of both arches were taken, followed by digital mock-up planning using Exo-CAD software. The mock-up was fabricated using a computer-aided milling machine with acrylic material (Figure 1).

In our case, the overbite was above 5 mm indicating room for incisal reduction. The main aim of the treatment was to close the space in the anterior teeth while decreasing the proclination of these teeth. The width and the height changes in teeth required to achieve this aim needed to be such that the proportionality and alignment of the teeth with the adjacent teeth and the face were maintained. Space closure of 0.5 mm to 0.75 mm on the mesial and distal aspect of the anterior teeth was required to be achieved. Using indices, Golden Proportion, and Berry’s Biometric Index, the mesiodistal width of the veneer to be prepared was determined. Once the mesiodistal width was determined, the width-to-height ratio was used to determine the height of each veneer required.

**Figure 2:**
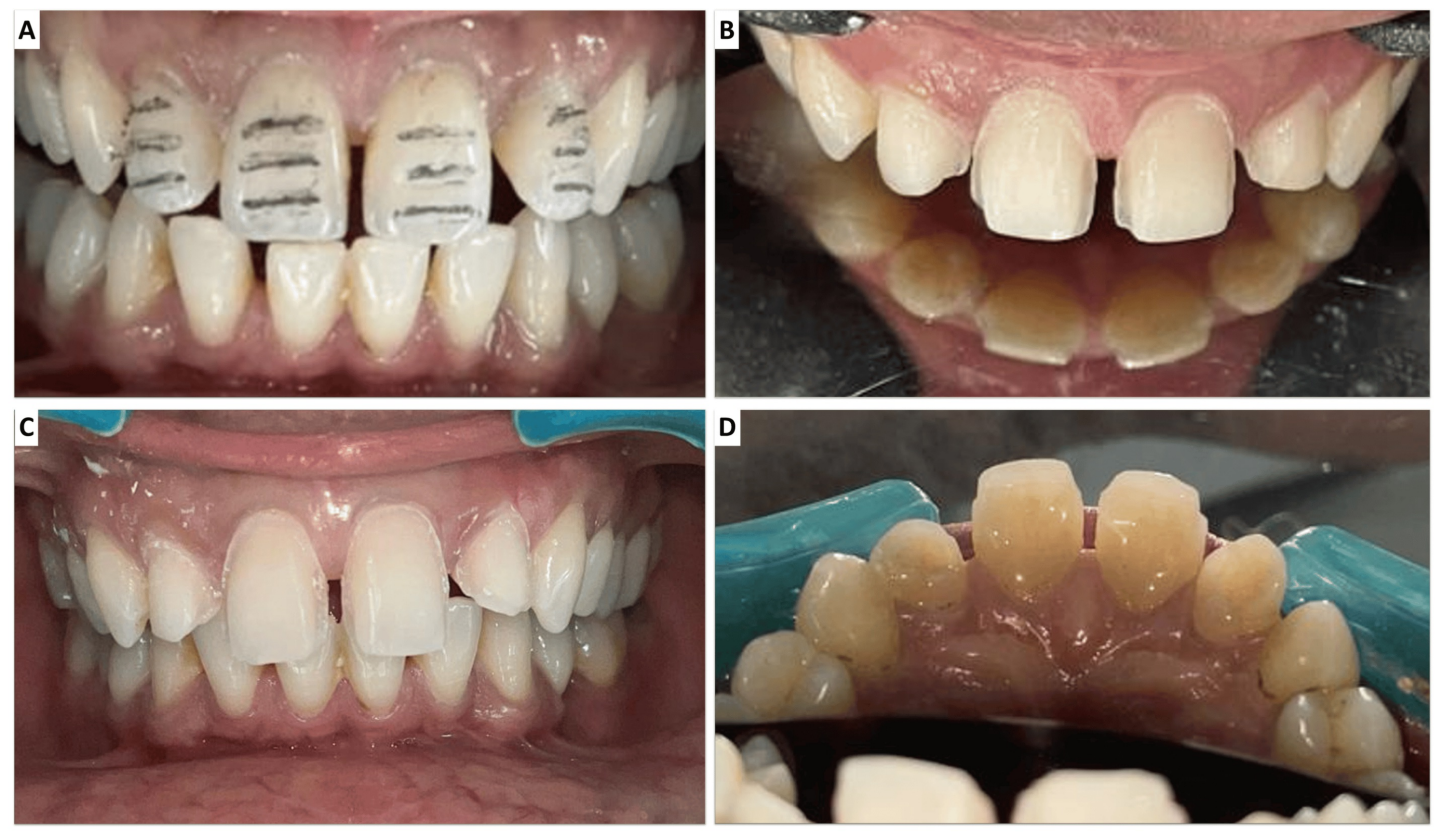
Tooth preparation steps depicting (A) depth cut, (B) labial preparation, (C) incisal reduction, and (D) palatal chamfer preparation.

The width-to-height ratio states that the ratio of the mesiodistal width of the tooth to the cervico-incisal height should range from 0.75 to 0.8. Depth cuts were given in the incisal middle cervical third with a bi-planar direction. A depth of 1.5 mm was achieved. Labial reduction of 1.5 mm was done to maintain the majority of the preparation in the enamel to ensure good bond strength. The margins of the veneers were strictly in the enamel to achieve adequate enamel bonding and avoid marginal disintegration and leakage. Tooth preparation involved minimal reduction of the enamel, maintaining supragingival margins with a radial shoulder design for optimal material thickness and strength (Figure 2).

Supragingival margins were maintained. The radial shoulder margin was given for enough thickness of material and structural durability. Incisal reduction of 2-2.5 mm was performed keeping the width-to-height ratio in mind. Proximal margins were not extended all the way to the palatal surface. This provided bonding to the palatal enamel and an easy path of insertion for the individual veneer. Embrasures were maintained to avoid a bulky look for the anterior teeth. The incisal step between the central and lateral incisor was maintained at 1-1.5 mm to achieve a feminine appearance. Line angles were kept rounded, and mesiodistal inclinations were given on the proximal surface of the tooth to give an illusion of narrow teeth.

**Figure 3:**
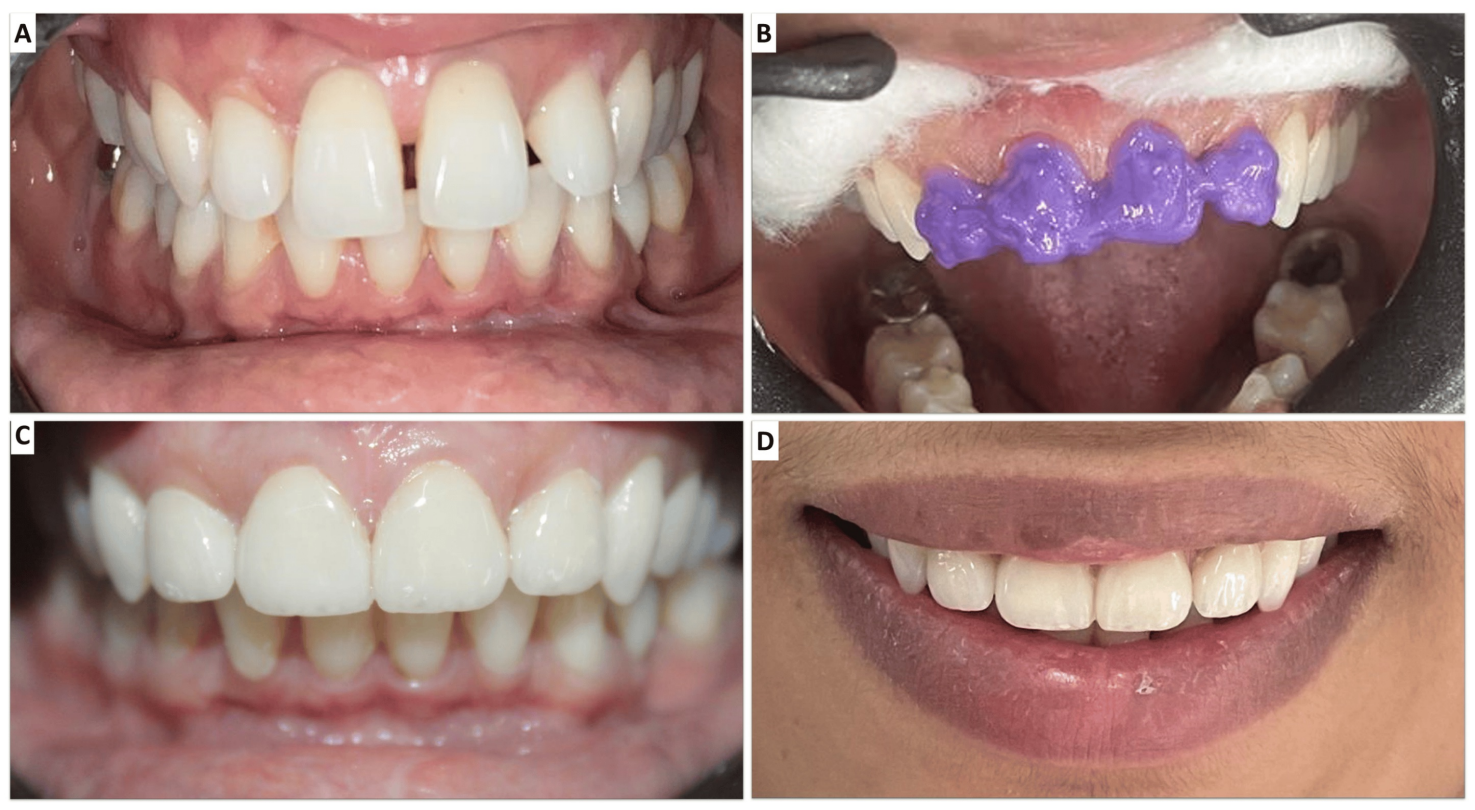
(A) Preoperative image. (B) Acid etching of the maxillary incisors. (C) Cementation of the final veneers. (D) Post-treatment smile.

Following tooth preparation, impressions were recorded using a two-step impression technique with medium and light body materials. Temporary restorations were fabricated using dual-cure acrylic material. The final veneers were milled using IPS e-max lithium disilicate glass-ceramic (Ivoclar Vivadent), known for its strength, translucency, and bonding properties. The veneers were bonded to the tooth structure using a standard etching and bonding protocol. The tooth surfaces were etched with 37% phosphoric acid, and the veneer intaglio surfaces were treated with hydrofluoric acid for 90 seconds before bonding with resin cement. The veneers were placed sequentially, starting with the central incisors, followed by the lateral incisors (Figure 3). Excess cement was removed after tack curing, and final curing was performed for 60 seconds on each tooth.

**Figure 4:**
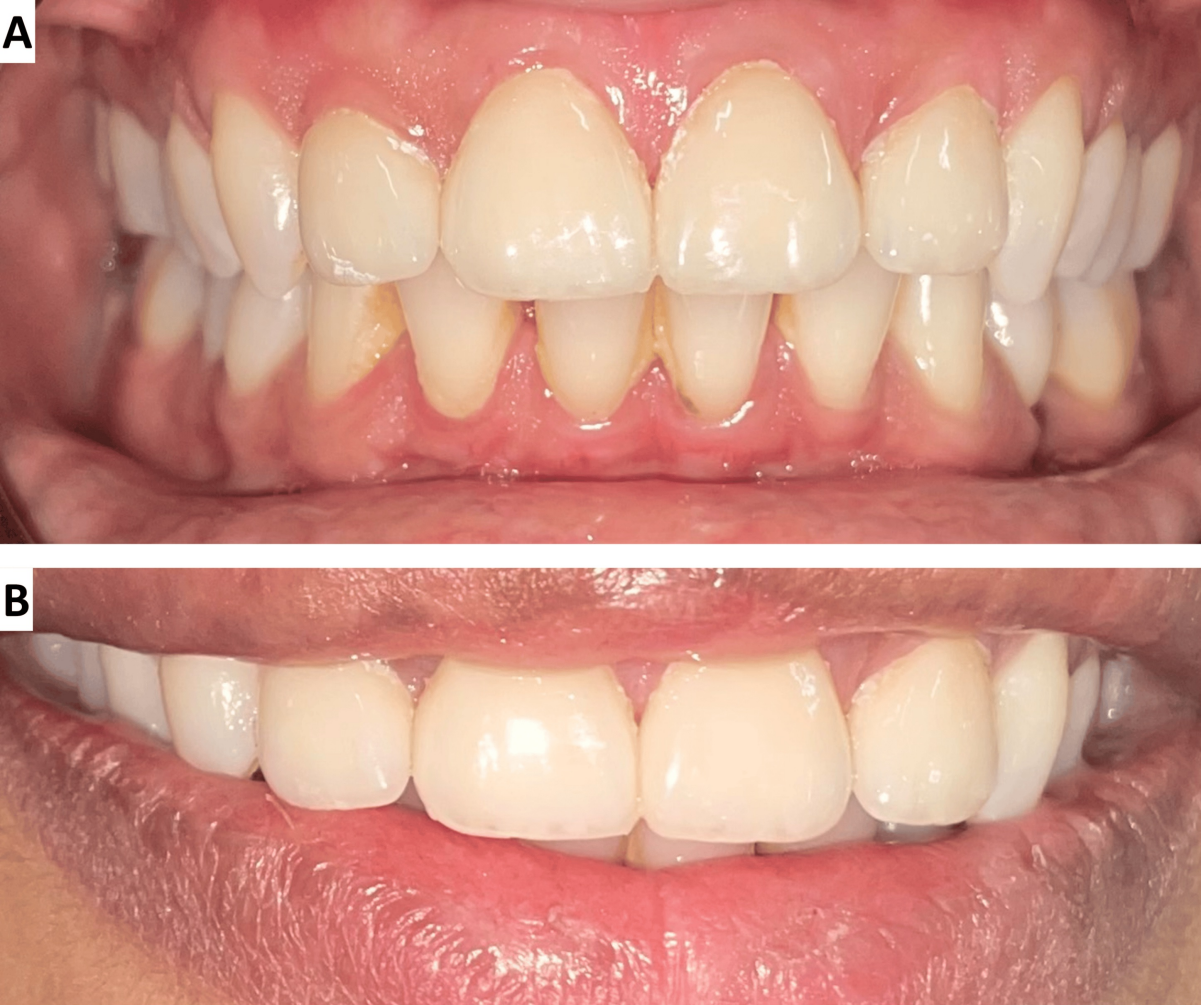
Sixth-month follow-up image of (A) teeth in occlusion and (B) smile.

Occlusal adjustments were made to ensure no interferences in lateral or protrusive movements. Anterior guidance and phonetics were checked by having the patient pronounce “M,” “S,” and “F” sounds to evaluate lip position, incisal guidance, and closest speaking space. Post-treatment evaluation showed a significant improvement in the aesthetics and alignment of the upper anterior teeth. The patient exhibited a straighter profile with reduced proclination and a well-balanced smile. Sixth-month follow-up showed stable results, with no signs of debonding, gingival inflammation, or marginal discoloration (Figure 4). The patient expressed satisfaction with the overall outcome.

## Discussion

The management of anterior spacing and proclination presents a complex challenge, particularly when a patient seeks an aesthetic solution without undergoing orthodontic treatment. In this case, lithium disilicate glass-ceramic laminate veneers were selected as the treatment of choice due to their minimally invasive nature, ability to improve dental aesthetics, and long-term durability. The veneers allowed for the closure of diastemas, correction of tooth alignment, and enhancement of the patient’s smile, all the while preserving a significant amount of natural tooth structure.

Lithium disilicate glass-ceramic laminate veneers have consistently demonstrated their utility in achieving high aesthetic results with minimal invasiveness [2]. Studies have shown that these restorations can effectively mimic the translucency, surface texture, and natural appearance of enamel, making them an ideal option for anterior teeth [5,6]. The use of IPS e-max veneers, as in this case, offers additional advantages due to the material’s high flexural strength and optical properties. Lithium disilicate, the material used in IPS e-max veneers, provides superior durability while allowing for conservative tooth preparation [7]. Moreover, their ability to be etched and bonded to enamel further enhances their retention and longevity.

One of the key considerations in the aesthetic restoration of anterior teeth is the use of mathematical principles to guide tooth proportions. In this case, both Berry’s Biometric Index and the Golden Proportion were employed to determine the ideal mesiodistal width and height of the veneers. Studies have supported the use of these indices in smile design, highlighting their importance in achieving facial harmony and optimal tooth proportions [4,8]. The Golden Proportion, which advocates for a 62% width ratio between adjacent teeth, has been particularly noted for creating aesthetically pleasing outcomes [9]. By utilizing these proportions, the treatment in this case ensured that the restored teeth not only closed the gaps but also maintained balance with the patient’s facial features.

Another important aspect of this treatment was ensuring the correct management of occlusion. Proper anterior guidance and occlusal harmony were critical to the success of the veneers in both functional and aesthetic aspects [10]. The use of digital mock-ups allowed for precise planning of tooth dimensions, occlusion, and aesthetic outcomes. Digital tools such as Exo-CAD have revolutionized veneer treatment planning by enabling clinicians to visualize and adjust the final outcome before the actual treatment is initiated [11].

Veneer preparation design is another factor that plays a significant role in the longevity and success of the restoration. In this case, a semi-invasive preparation with a palatal chamfer was selected. Such preparations in enamel allow for superior bonding and reduce the likelihood of debonding over time compared to feather-edge or butt joint designs [12]. The decision to maintain the preparation primarily in enamel has been reinforced by studies that show significantly better bonding strength to enamel than to dentin, contributing to the long-term success of veneers.

Follow-up care and regular monitoring of veneer restorations are essential for maintaining both function and aesthetics. In this case, a sixth-month follow-up showed stable results with no signs of debonding, discoloration, or gingival inflammation, consistent with existing literature on the long-term success rates of lithium disilicate glass-ceramic laminate veneers. Additionally, patient satisfaction with the aesthetic outcome was high, further emphasizing the positive psychological impact of such restorative treatments.

However, it is important to acknowledge that veneers, while highly aesthetic and conservative, are not without limitations. The potential for veneer fracture, debonding, or marginal discoloration exists, particularly if occlusal forces are not adequately managed or if the bonding procedure is not properly executed [13]. Additionally, patient selection is crucial, as excessive parafunctional habits or significant occlusal discrepancies may predispose the veneers to early failure [14]. Another drawback of the present case was the selection of supragingival radial shoulder margin. Given the aesthetic requirement, a subgingival chamfer margin would have been more appropriate for superior aesthetic outcome.

Overall, the present case demonstrates the effective use of lithium disilicate glass-ceramic laminate veneers in addressing both aesthetic and functional concerns in a patient with anterior spacing and proclination. The combination of minimally invasive preparation, the application of mathematical indices for smile design, and the use of high-quality materials such as IPS e-max veneers contributed to a successful and aesthetically pleasing outcome. Future studies and long-term follow-ups are recommended to further substantiate the benefits of these restorations in various clinical scenarios.

## Conclusions

Lithium disilicate glass-ceramic laminate veneers offer an effective and minimally invasive solution for addressing aesthetic concerns such as spacing and proclination in the anterior teeth. The use of modern materials such as IPS e-max, along with precise digital planning and adherence to aesthetic principles, ensures both functional and aesthetic success. Long-term follow-up and proper case selection are essential to maximize the longevity and predictability of these restorations.
